# Benzonatate as a local anesthetic

**DOI:** 10.1371/journal.pone.0284401

**Published:** 2023-04-12

**Authors:** Anna McGuire, Claire A. Ostertag-Hill, Gil Aizik, Yang Li, Daniel S. Kohane

**Affiliations:** 1 Laboratory for Biomaterials and Drug Delivery, Boston Children’s Hospital, Harvard Medical School, Harvard Institutes of Medicine, Boston, Massachusetts, United States of America; 2 Department of Surgery, Boston Children’s Hospital, Harvard Medical School, Boston, Massachusetts, United States of America; 3 Department of Anesthesiology, Boston Children’s Hospital, Harvard Medical School, Boston, Massachusetts, United States of America; Wright State University, UNITED STATES

## Abstract

**Introduction:**

Benzonatate is an FDA-approved antitussive agent that resembles tetracaine, procaine, and cocaine in its chemical structure. Based on structural similarities to known local anesthetics and recent findings of benzonatate exerting local anesthetic-like effects on voltage-gated sodium channels *in vitro*, we hypothesized that benzonatate will act as a local anesthetic to yield peripheral nerve blockade.

**Methods:**

Benzonatate was injected at the sciatic nerve of Sprague-Dawley rats. Sensory and motor blockade were assessed using a modified hot plate test and a weight-bearing test, respectively. Additionally, the effect of co-injection with tetrodotoxin and Tween 80 (a chemical permeation enhancer) was examined. Myotoxicity of benzonatate was assessed *in vivo* by histological analysis.

**Results:**

Benzonatate produced a concentration-dependent sensory and motor nerve blockade with no appreciable systemic effects. Co-injection with tetrodotoxin or Tween 80 produced prolongation of sensory nerve blockade. Histologic assessment showed significant inflammation and myotoxicity from benzonatate injection, even at low concentrations.

**Conclusion:**

This study demonstrates that benzonatate does act as a local anesthetic at the peripheral nerve, with sensory and motor nerve blockade. Benzonatate interacts with tetrodotoxin and Tween 80 to prolong nerve blockade. However, benzonatate causes significant myotoxicity, even at subtherapeutic concentrations.

## Introduction

**Fig 1:**
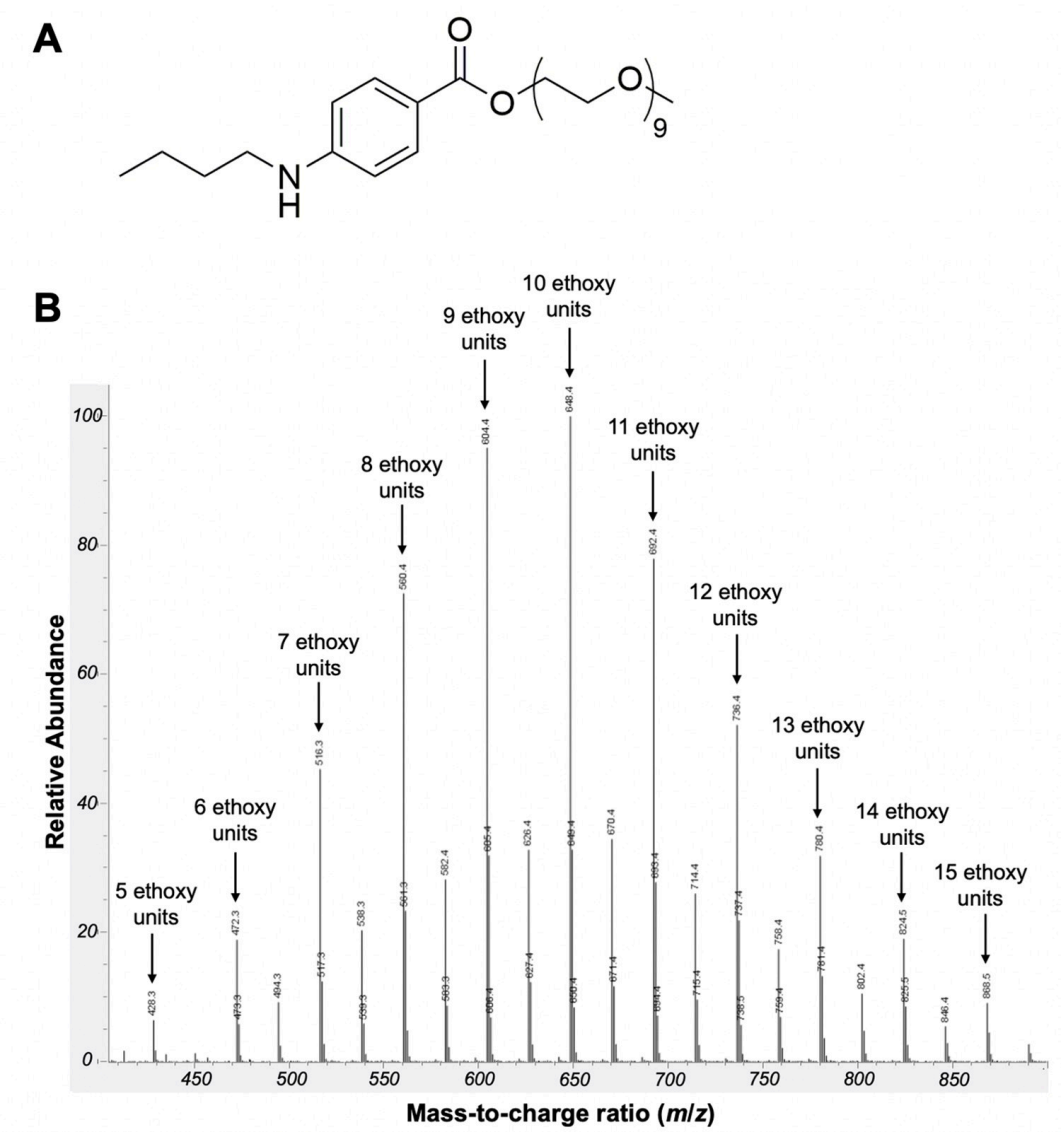
Characterization of benzonatate. (A) chemical structure of benzonatate (B) LC-MS of benzonatate used in this study.

Benzonatate (Fig 1A), an orally administrated non-narcotic cough suppressant first approved in 1958 [1], is chemically related to amino ester local anesthetics such as tetracaine. Cough suppression is mediated by inhibition of pulmonary stretch receptors, possibly through inhibition of voltage-gated sodium channels, including Nav1.7, in a reversible and concentration-dependent manner [2]. Since conventional local anesthetics (CLAs) and site 1 sodium channel blockers (S1SCBs, such as tetrodotoxin, TTX) provide local anesthesia *via* inhibition of these voltage-gated sodium channels [3], benzonatate could act as a local anesthetic. This potential is supported by benzonatate’s ability to provide rapid topical oropharyngeal anesthesia for awake intubation [4]. However, benzonatate is not currently being used as a local anesthetic for peripheral nerve blockade.

Here, we examined the hypothesis that benzonatate can act as a local anesthetic to provide peripheral nerve blockade. We developed a dose-response curve for the efficacy and safety of benzonatate. We further examined the interactions of benzonatate with other compounds that are known to enhance nerve block by local anesthetics. CLAs and S1SCBs in combination have a markedly prolonged effect, in part due to blockade at different locations on the same voltage-gated sodium channel. We combined benzonatate with TTX, a very hydrophilic small molecule that binds to site 1 on the extracellular part of the sodium channel [5,6]. Given the structural similarity of benzonatate to CLAs, one would expect nerve block from benzonatate to be markedly enhanced by TTX. We also examined the effect of a chemical permeation enhancer (CPE), Tween 80 (T80). We have previously shown T80 to markedly increase the duration of nerve blockade by TTX but not the CLA bupivacaine [5], presumably because the former had difficulty reaching the neuronal surface due to its hydrophilicity, while the latter does not, being amphiphilic.

## Methods

### Preparation of test solutions

Benzonatate (Sigma-Aldrich, St. Louis, MO) was dissolved in ultrapure water to the desired concentration (range 5.4–198.8 mM, 3.25–120 mg/mL) with a standard injection volume of 0.3 mL. Benzonatate was characterized by liquid chromatography–mass spectrometry (LC-MS; Agilent Technologies, Santa Clara, CA).

Bupivacaine hydrochloride salt (Sigma-Aldrich, St. Louis, MO) and TTX (citrate free, >98% purity, Abcam, Cambridge, MA) were prepared in normal saline solution (0.9% NaCl) individually and in combination with benzonatate (for TTX only). The TTX (30 μM) concentration was chosen to be near the lower inflection point of previously established dose-response curves, allowing for clear detection of any prolongation of nerve blockade [5]. Tween 80 (T80) (Sigma-Aldrich, St. Louis, MO) was diluted with 0.9% NaCl to a standard injection volume of 0.3 mL (concentration 23 mM) with and without benzonatate as above (Table 1). Bupivacaine concentrations (2.1 mM and 15.4 mM) were selected for histological analyses to permit comparisons of equieffective lower and higher concentrations of benzonatate and bupivacaine [5]. The higher concentration selected corresponds to a relatively high concentration of bupivacaine used clinically (15.4 mM, 0.5% w/v).

**Table 1:** Formulations injected at the sciatic nerve.

Benzonatate (mM)	Co-administered with	n	Sensory Block Rate (%)	Sensory Block Duration (min)	Motor Block Duration (min)
5.4	-	4	25.0	0 (0, 18.5)	43.7 (32.1, 50.9)
12.4	-	12	66.7	49.0 (0, 76.7)	59.3 (44.7, 88.3)
24.8	-	4	75.0	84.5 (48.4, 112.7)	91.2 (57.3, 117.7)
49.7	-	4	100.0	149.9 (133.8, 151.3)	149.0 (134.9, 151.8)
74.5	-	4	100.0	135.0 (123.3, 141.5)	130.6 (118.5, 138.0)
99.4	-	8	100.0	157.4 (148.4, 166.2)	159.9 (148.6, 168.3)
149.1	-	4	100.0	197.1 (196.5, 197.7)	224.8 (217.7, 225.5)
198.8	-	4	100.0	225.4 (215.1, 231.2)	277.3 (231.0, 358.7)
-	30 μM TTX	8	25.0	0 (0, 12.3)	0 (0, 40.6)
12.4	30 μM TTX	8	100.0	77.9 (74.7, 141.7)	84.6 (77.3, 180.4)
-	23 mM T80	8	0.0	0 (0, 0)	0 (0, 0)
12.4	23 mM T80	8	100.0	77.0 (76.1, 104.9)	107.0 (105.3, 133.5)

- Indicates that agent was not administered.

Sensory and motor block durations are represented as medians with 25^th^ and 75^th^ percentiles.

### Animal care

Adult male Sprague-Dawley rats (300–400 g) were obtained from Charles River Laboratories (Wilmington, MA). Animals were housed in groups of two per cage on a 7 am to 7 pm light/dark cycle and fed with standard laboratory chow and water *ad libitum*. All animals were cared for in accordance with protocols approved by the Animal Care and Use Committee at Boston Children’s Hospital, as well as the Guide for the Care and Use of Laboratory Animals of the US National Research Council.

### Sciatic nerve blockade technique

Rats were anesthetized using isoflurane. A 23-gauge needle was introduced posteromedial to the greater trochanter, and upon contact with the bone, 0.3 mL of the test compound or compounds were injected, depositing the injectate over the sciatic nerve as previously described [7]. All injections were performed on the left leg with the right leg serving as an untreated, negative control. Experimenters performing the injections have demonstrated >99% successful sciatic nerve blocks with 0.1 mL of 0.5% bupivacaine prior to beginning the experiments reported in the study, i.e., zero duration blocks in this study are a reflection of local anesthetic efficacy, not operator error (“missing”). Injection with normal saline, the vehicle used in this study, has been repeatedly shown not to produce nerve blockade [8,9].

### Sciatic nerve blockade assessment

In all experiments, the experimenter assessing sciatic nerve block duration was blinded to the treatment the animal had received. The presence and extent of sciatic sensory nerve blockade (thermal nociception) was assessed using a modified hot plate test as previously described [7,10]. Animals are held above the hot plate by the experimenter and hind paws were exposed in sequence (left then right) in triplicate to a 56°C hot plate (model 39D Hot Plate Analgesia meter; IITC Inc., Woodland Hills, CA). The time (thermal latency) until the animal withdraws its hind paw was measured. If the animal did not withdraw the hind paw after 12 s, the experimenter removed the hind paw to prevent injury or the development of hyperalgesia. Animals received a 10 s pause between each assessment. Testing was conducted at the following intervals after injection: 30 min, 60 min, then hourly until full nerve block resolution, as indicated by reaching a previously established baseline thermal latency of 2 s [7]. The duration of the thermal nociceptive block (sensory block) was defined as the time (minutes) required for thermal latency to resolve to an average value less than 7 s. The duration of block was calculated as follows: [Last latency above 7–7] / [Latency last latency above 7 –First latency below 7] * [Timepoint of First latency below 7 –Timepoint of Last Latency above 7] + Timepoint of Last Latency above 7. For example, if an individual rat has a latency of 10 s at 30 min and 4 s at 60 min, the duration of block is [10–7]/[10–4] * [60–30] + 30 = 45 min. The duration of block for a group of animals is then expressed as the median and 25^th^ and 75^th^ percentiles of durations of block obtained in this way. Seven seconds corresponds to the midpoint between the baseline thermal latency of approximately two seconds in adult rats and the maximal latency of 12 s. A successful nerve block was defined as a thermal latency greater than 7 s averaged across the triplicate measures.

Motor blockade was assessed using the extensor postural thrust (EPT) test. The animal’s hind paws were suspended over a digital balance in sequence (left then right) and the maximum weight that the animal could bear was measured in triplicate. A motor block was defined as the inability to bear half-maximal body weight. The duration of the motor blockade was defined as the time (minutes) required for weight-bearing to return halfway to normal from the maximal block.

The examiner was blinded as to the treatment group of any individual rat.

### Tissue harvesting and histology

Animals were euthanized with carbon dioxide four days after injection, the time point at which local anesthetic inflammation and toxicity is expected [11]. The sciatic nerve and surrounding tissues were harvested, fixed in 10% neutral buffered formalin, embedded in paraffin, sectioned, and stained with hematoxylin and eosin using standard techniques.

Muscle samples were scored for inflammation (0–4) and myotoxicity (0–6) as previously described [12–14]. The inflammation score reflects a subjective assessment of the severity of inflammation. The myotoxicity score considers two separate but related hallmarks of local anesthetic myotoxicity, specifically nuclear internalization and regeneration. Nuclear internalization is characterized by normal myocyte size and cytoplasm chromicity but with nuclei located away from their normal peripheral location. Regenerating myocytes are characterized by a shrunken appearance with basophilic cytoplasm. Scoring occurred as follows; 0 = normal; 1 = perifascicular internalization; 2 = deep internalization (>5 cell layers); 3 = perifascicular regeneration; 4 = deep regeneration; 5 = hemifascicular regeneration; 6 = holofascicular regeneration. The evaluator was blinded to the nature of the samples.

### Statistical analysis

**Fig 2:**
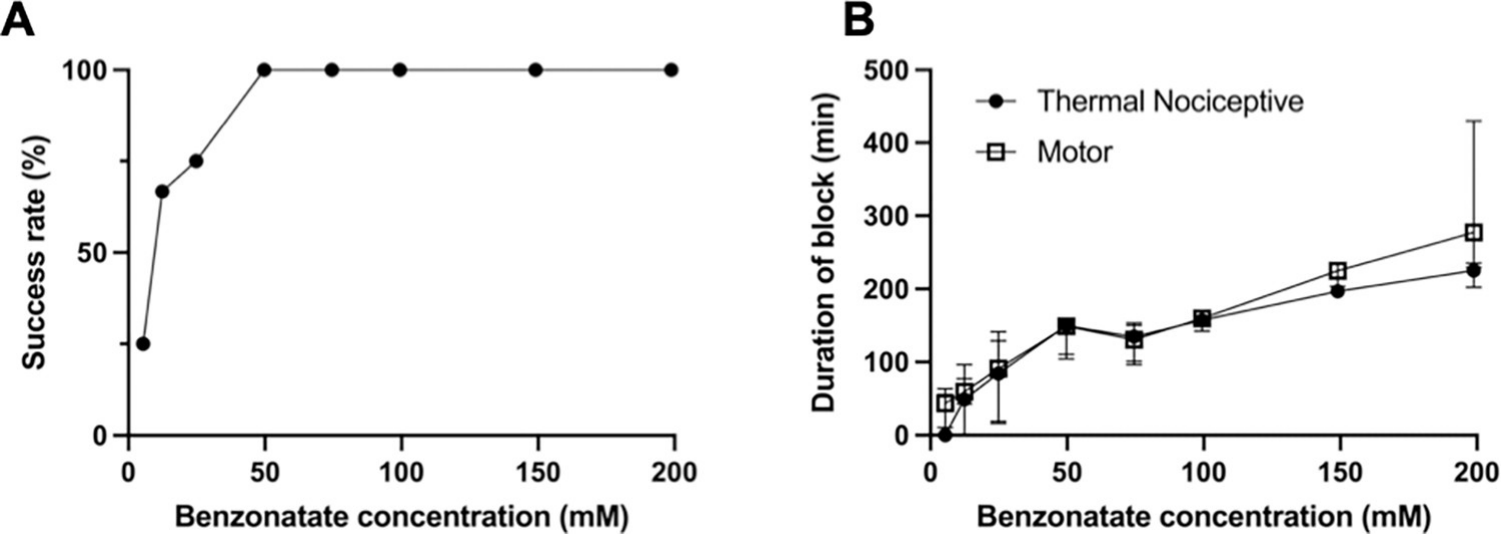
Effect of benzonatate concentration on nerve blockade. (A) Frequency of successful thermal nociceptive block with varying concentrations of benzonatate. n = 4 per group except n = 8 for 99.4 mM and n = 12 for 12.4 mM. (B) Duration of nerve blockade from benzonatate. Data are medians with 25^th^ and 75^th^ percentiles, n = 4 except n = 8 for 99.4mM and n = 12 for 12.4 mM. p>0.05 for comparison of durations of sensory and motor block at each concentration by the Wilcoxon signed-rank test.

Neurobehavioral data are presented as medians with 25^th^ and 75^th^ percentiles. The median durations of block (e.g., Fig 2B) include injections resulting in no measurable block. Inflammation and myotoxicity scores are represented as medians with 25^th^ and 75^th^ percentiles. Sensory and motor block duration of animals receiving benzonatate were compared by the Wilcoxon signed-rank test. Inflammation and myotoxicity scores are compared by the Mann-Whitney U test. Statistical significance was defined as p < 0.05. Neurobehavioral data comparing various agents (benzonatate, TTX, T80, and co-administrations of these compounds) were analyzed using a Kruskal-Wallis Test with post-hoc Mann-Whitney U Test pair-wise comparisons with Bonferroni correction relative to benzonatate alone. For this comparison, the p-value required for statistical significance was 0.0125 (due to the number of comparisons that were made).

## Results

### Characterization and composition of benzonatate

Benzonatate (2, 5, 8, 11, 14, 17, 20, 23, 26-nonaoxaoctacosan-28-yl p-(butylamino) benzoate) including the compound available for clinical use [15], can have a varying number of repeated ethoxy units (Fig 1A) [2]; hydrophobicity increases with increasing number of ethoxy units [2]. LC-MS analysis of the benzonatate used in this study (Sigma-Aldrich) revealed a mixture of compounds with 5–15 ethoxy units (Fig 1B). For the purposes of this work, we have assumed a molecular weight of 603.7 g/mol (i.e., nine repeated ethoxy units) per manufacturer specifications.

### Sciatic nerve blockade with benzonatate

The incidence of successful (non-zero duration) nerve blocks (see Methods for definition) increased with the concentration of benzonatate injected at the left sciatic nerve (Fig 2A, Table 1). Zero-duration nerve blocks occurred only at benzonatate concentrations below 49.7 mM. Similarly, there was a concentration-dependent increase in the median duration of nerve blockade (Fig 2B; data include zero-duration blocks). There was no statistically significant difference between the durations of sensory and motor nerve blockade at any concentration of benzonatate. The highest concentration tested (198.8 mM) produced a median sensory nerve blockade 225.4 (215.1, 231.2) minutes and motor nerve blockade of 277.3 (231.0, 358.7) minutes.

No neurobehavioral deficits were detected in the contralateral (uninjected) limb in any animal injected with benzonatate. Deficits in the contralateral extremity are a sign of systemic drug distribution. All nerve blocks resolved completely as indicated by return to a latency of 2 s.

### Co-administration of benzonatate and tetrodotoxin

12.4 mM benzonatate was selected to examine the effect of co-injection with TTX, because the modest effect of that concentration of benzonatate (66.7% successful block, 49 (0, 76.7) minutes sensory block; Fig 2) would make it easy to detect enhancement in performance. The selected concentration of TTX (30 μM) was based on previous observations that this concentration also had a modest effect and would similarly allow for detection of increases in nerve blockade. [5,7] Injection of 0.3 mL of 30 μM TTX produced 25% successful blocks, with sensory block lasting a median of 0 (0, 12.3) minutes and motor block lasting a median of 0 (0, 40.6) minutes (n = 8). There was no statistically significant difference between the durations of sensory and motor blockade from 30 μM TTX (p = 0.75).

**Fig 3:**
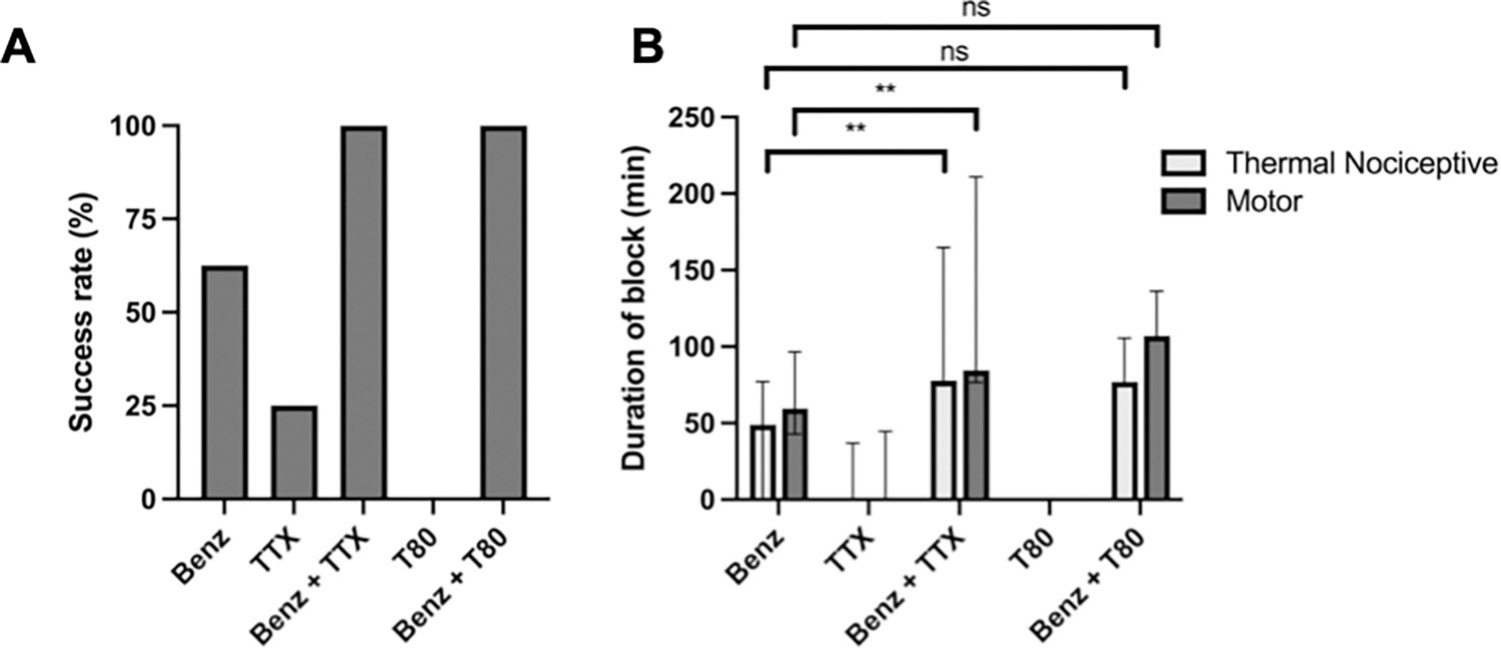
Effect of co-administration of benzonatate (Benz, 12.4 mM) with TTX (30 μM) or T80 (23 mM). (A) frequency of successful sensory nerve block (B) duration of sciatic sensory and motor nerve block. Data are medians with 25^th^ and 75^th^ percentiles, n = 8 per group except benzonatate administration with n = 12 (note that administration of T80 alone resulted in no sensory or motor block for all animals). Duration of nerve block were compared by a Kruskal-Wallis test (p = 0.0001) with post-hoc pairwise comparison with Bonferroni correction (p<0.01 for sensory and motor blockade following co-administration of benzonatate and TTX compared to benzonatate alone).

Co-injection of benzonatate with TTX increased the successful block rate to 100% (n = 8) (Fig 3A) with a 1.5-fold prolongation of sensory blockade to a median of 77.9.0 (74.7, 141.7) minutes (Fig 3B; p = 0.0004 compared to benzonatate alone). The duration of motor blockade was significantly prolonged with co-injection of TTX (p = 0.003) to a median duration of 84.6 (77.3, 180.4) minutes (Fig 3B). There was no statistically significant difference between the durations of sensory and motor nerve blockade from benzonatate co-injected with TTX (p = 0.38).

### Co-administration of benzonatate and Tween 80

A variety of CPEs have been demonstrated to cause a concentration-dependent increase in frequency and duration of block with TTX, but not with bupivacaine [5]. We selected the nonionic surfactant T80 due to its low tissue toxicity [5]. T80 itself did not cause detectable sensory or motor nerve blockade at the concentration used in this study, 23 mM (Fig 3A) [5].

T80 was co-injected with 12.4 mM benzonatate (this concentration selected for the same reason as for co-injection with TTX). Co-injection of 12.4 mM benzonatate and 23 mM T80 resulted in 100% of animals having sensory blockade (compared to 66.7% of animals with benzonatate alone; Fig 3A). Although the duration of sensory nerve blockade was prolonged 1.5-fold, to a median of 77.0 (76.1, 104.9) minutes and motor blockade to 107.0 (105.3, 133.5) minutes, these increases were not statistically significantly different from benzonatate alone (Fig 3B; p = 0.07 for sensory nerve blockade, p = 0.12 for motor nerve blockade). Motor block duration was 1.4-fold longer than sensory block duration with the co-injection of benzonatate and T80 (p = 0.01).

### Tissue toxicity of benzonatate

Tissue toxicity of benzonatate was assessed and compared to that from the commonly used CLA bupivacaine, at concentrations that were equieffective in duration of sensory block. 12.4 mM benzonatate (sensory block: 49.0 (0, 76.7) minutes) was compared to 2.1 mM bupivacaine (sensory block: 45.6 (44.5, 60.5) minutes), p = 0.85 for the comparison of the duration in the two groups. 99.4 mM benzonatate (sensory block: 157.4 (148.4, 166.2) minutes) was compared to 15.4 mM bupivacaine (sensory block: 154.3 (142.8, 154.3) minutes), p = 0.68.

**Fig 4:**
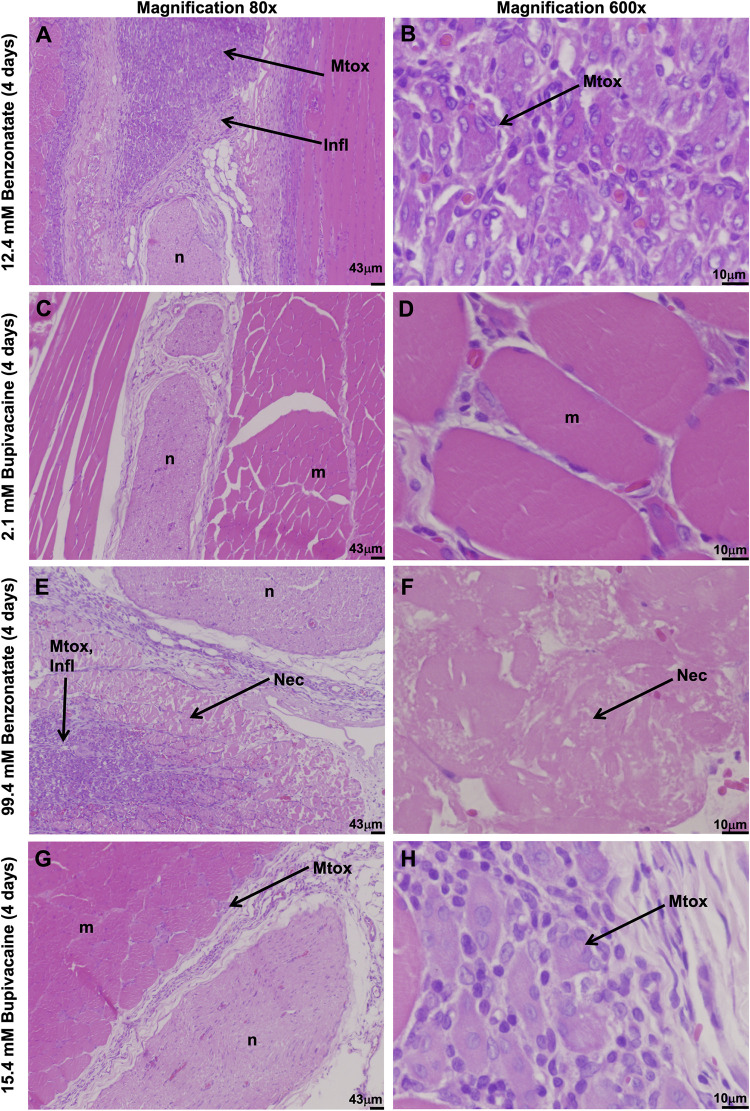
Representative light microscopy of hematoxylin/eosin-stained sections of nerves (n), muscles (m), and surrounding tissues at the site of injection. Tissues were collected 4 days after injection with equieffective concentrations of benzonatate and bupivacaine. Infl: Inflammation. Mtox: Myotoxicity. Nec: Necrosis.

**Fig 5:**
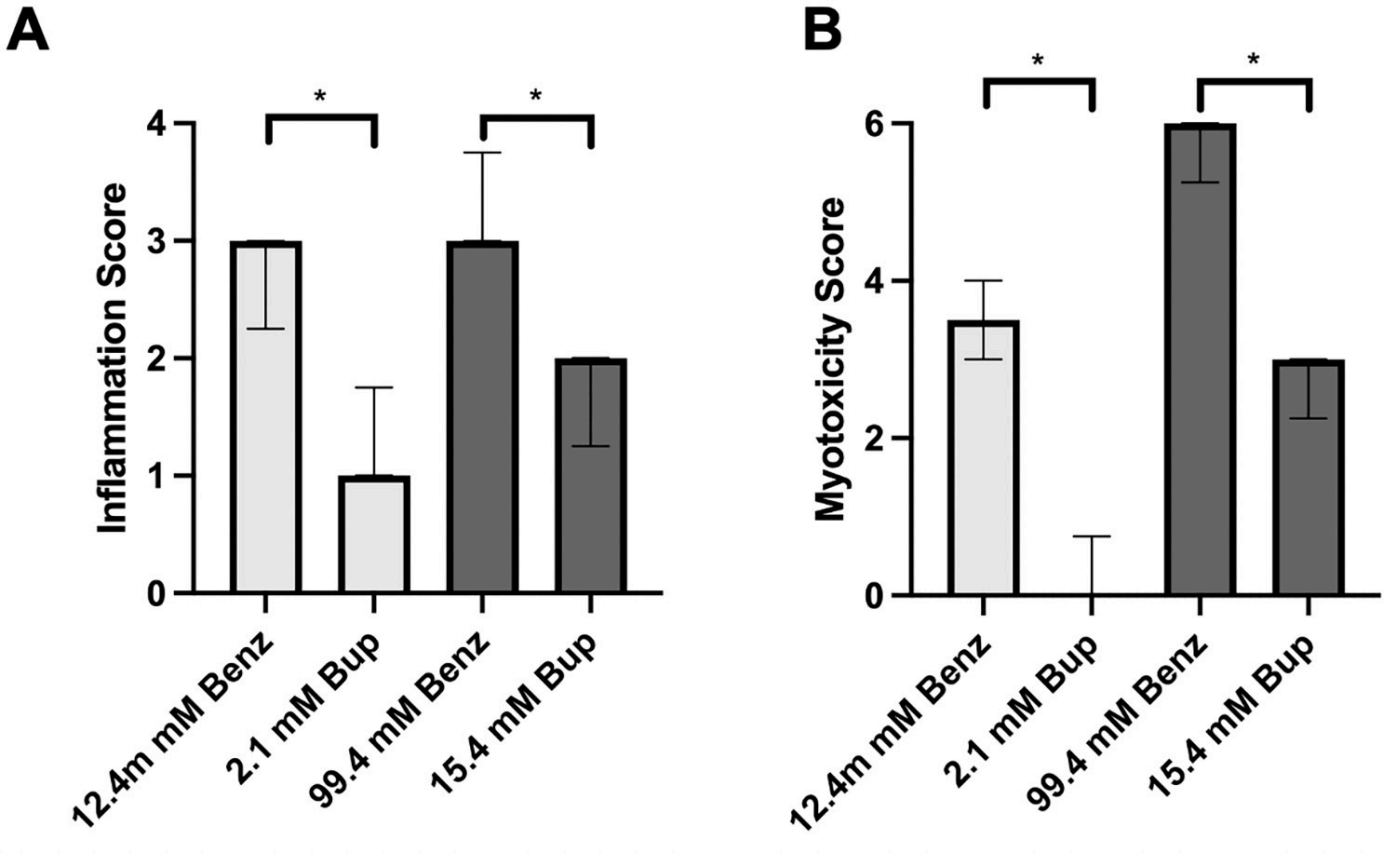
Quantification of tissue reaction. (A) Inflammation and (B) myotoxicity scores of animals injected at the sciatic nerve with benzonatate and bupivacaine. Equieffective concentrations have the same shading. Data are medians with 25^th^ and 75^th^ percentiles, n = 4 for all groups. * p < 0.05 for the comparison of equieffective concentrations by Mann-Whitney U test.

Four days after injection, animals were euthanized, and nerve, muscle, and surrounding tissues were harvested at the site of injection and processed into hematoxylin and eosin-stained sections. All animals injected with benzonatate demonstrated considerable inflammation and myotoxicity on histologic examination. Tissue reaction to 12.4 mM benzonatate consisted of a mixed inflammatory infiltrate with neutrophils, lymphocytes, and macrophages (Fig 4A and 4B), corresponding to a median inflammation score of 3 (range 2–3) (Fig 5A). (A box-and-whiskers version of Fig 5 is provided in S1 Fig). Significant myotoxicity with perifascicular and deep regeneration was evident, reflected in a median myotoxicity score of 3.5 (range 3–4) (Fig 5B). By comparison, animals injected with an equieffective (2.1 mM) concentration of bupivacaine demonstrated only minimal inflammation (median inflammation score of 1, p = 0.03) with minimal to no myotoxicity (median myotoxicity score of 0, p = 0.02) (Figs 4C, 4D, 5A and 5B).

Animals injected with the higher concentration of benzonatate (99.4 mM) also showed considerable muscle inflammation (Fig 4E and 4F) with a median score of 3 (range 3–4) (Fig 5A). Myotoxicity involved over half of the muscle bundle (median myotoxicity score of 6) (Fig 5B). Additionally, there were large eosinophilic muscle cells with absent nuclei, consistent with muscle necrosis, in a perifascicular distribution, i.e., closest to the injection site (Fig 4F). The equieffective concentration of bupivacaine (15.4 mM) produced much less inflammation (p = 0.02) and myotoxicity (p = 0.02) with centralization of nuclei and only perifascicular muscle injury (Fig 4G and 4H).

## Discussion

We sought to investigate the potential of benzonatate, a non-narcotic cough suppressant approved by the FDA in 1958 [1], as a local anesthetic. Benzonatate, injected locally at the sciatic nerve of rats, produced concentration-dependent sensory and motor nerve blockade, confirming that benzonatate does act as a local anesthetic at peripheral nerve as hypothesized. We show that benzonatate can generate similar nerve block durations to those observed with clinically relevant concentrations of bupivacaine, a common clinically used local anesthetic agent, albeit with lesser potency. Neurobehavioral deficits of the contralateral limb, a finding that suggests systemic drug distribution, was not observed with benzonatate [12,14]. However, considerable myotoxicity was observed even at concentrations where there was little anesthetic effect.

The potential of benzonatate as a local anesthetic has been suggested [2], but not proven, based on its structural similarities to ester-type local anesthetics such as tetracaine, procaine, and cocaine, previous *in vitro* studies [2], and clinical observations of rapid oropharyngeal anesthesia by topical benzonatate [4]. This also is supported by 1 mM benzonatate causing nearly 100% inhibition of sodium currents in murine cell lines [2]. However, 10 mM benzonatate had a similar effect to 1 mM of tetracaine (i.e., it was less potent). We demonstrate similar findings *in vivo*: benzonatate is less potent than the commonly used amino-amide local anesthetic bupivacaine, as evidenced by the fact that ~6-fold higher concentrations of benzonatate were required to achieve the same durations of block as from bupivacaine (which is similar in potency to tetracaine) [16].

Benzonatate is a mixture of molecules with variation in the number of repeating ethoxy units between different manufacturers [2,15,17]. Since the number of ethoxy units could affect the pharmacological properties of benzonatate, a longer ethoxy chain could increase the hydrophobicity of the molecule, impacting tissue retention time, and ease of penetration through the epineurium, perineurium, and endoneurium to the neurons. The number of ethoxy units could also affect the affinity of benzonatate to its binding site on neuronal sodium channels, as suggested by the observation that benzonatate molecules with different number of ethoxy units produce varying levels of sodium channel inhibition *in vitro* [2].

Drug interactions leading to prolongation of nerve block occur among various combinations of local anesthetics acting through different mechanisms, including between CLAs and site 1 sodium channel blockers (*e*.*g*., TTX) [7,18]. Such interactions have also been observed between site 1 sodium channel blockers and amino amide quaternary lidocaine derivatives [19], vanilloid receptor agonists [20], tricyclic antidepressants [21], and adrenergic antagonists [22]. Based on the similarity of benzonatate’s physicochemical properties to those of CLAs, prolongation of local anesthetic effects could be expected with site 1 sodium channel blockers. Indeed, a 1.5-fold longer sensory nerve block (p = 0.0004) was observed following co-administration of benzonatate and TTX. Benzonatate might also prolong the effect of TTX, at least in part, by acting as a CPE, as it has a structural similarity to tetracaine, and tetracaine is known to act as a CPE [23].

CPEs enhance nerve block from TTX, a highly hydrophilic molecule, by improving its diffusion across biological barriers [5]. In contrast, CLAs such as bupivacaine, an amphiphilic molecule, cross biological barriers without difficulty and therefore do not benefit from CPEs. Our observation that T80 prolonged nerve blockade of benzonatate 2-fold was therefore unexpected. One potential mechanism by which T80 enhanced benzonatate blockade could be that benzonatate’s relatively high molecular weight (603 Da) compared to that of bupivacaine (288 Da), was a hindrance to flux across barriers to the neuronal surface.

Myotoxicity is common with local anesthetics [24]. The intensity of tissue reaction to local anesthetics are related to the administered concentration and duration of exposure [25–27]. Bupivacaine, a local anesthetic in current clinical use, is among the more myotoxic CLAs [13,28]. Benzonatate caused inflammation and myotoxicity to a much greater degree than equieffective concentrations of bupivacaine, including a relatively high but clinically used concentration of the latter (15.4 mM, 0.5% w/v). Tissue toxicity would likely curtail the clinical application of benzonatate in peripheral nerve blockade. However, although local anesthetics sometimes cause severe myotoxicity [29], this has not generated much clinical concern. For example, intramuscular local anesthetic injection into trigger points is standard in myofascial pain syndromes [30].

We have shown that benzonatate is an effective local anesthetic for peripheral nerve blockade and that it shares many of the drug interactions seen with CLAs. However, it does not have advantages over bupivacaine, a local anesthetic in common clinical use, in terms of anesthetic effect or biocompatibility.

## Supporting information

S1 Fig Quantification of tissue reaction represented by a box and whisker plot. (a) Inflammation and (b) myotoxicity scores of animals injected at the sciatic nerve with benzonatate and bupivacaine. Equieffective concentrations have the same shading. Data are medians with 25^th^ and 75^th^ percentiles, n = 4 for all groups. Note that the median value (bar) overlaps with the 25^th^ or 75^th^ percentiles (box) for multiple data points. * P < 0.05 for the comparison of equieffective concentrations by Mann-Whitney U test.
(DOCX)

S1 Table Formulations injected at the sciatic nerve. (DOCX)

S2 Table Tissue toxicity of benzonatate: Inflammation and myotoxicity scores. (DOCX)
