# Supplementary material for: Benzonatate as a local anesthetic
Source: PLoS One. 2023 Apr 12;18(4):e0284401. doi: 10.1371/journal.pone.0284401 (PMC10096445; doi:10.1371/journal.pone.0284401)
Supplement: S1 Fig — (a) Inflammation and (b) myotoxicity scores of animals injected at the sciatic nerve with benzonatate and bupivacaine. Equieffective concentrations have the same shading. Data are medians with 25th and 75th percentiles, n = 4 for all groups. Note that the median value (bar) overlaps with the 25th or 75th percentiles (box) for multiple data points. * P < 0.05 for the comparison of equieffective concentrations by Mann-Whitney U test. (DOCX) [file pone.0284401.s001.docx]

**Supporting Information**

**Benzonatate as a local anesthetic**

Anna McGuire, ^1, 2¶^ Claire A. Ostertag-Hill,^1, 2¶^ Gil Aizik,^1, 3^ Yang Li, ^1, 3^ Daniel S. Kohane^1,3*^

^1^Laboratory for Biomaterials and Drug Delivery, Boston Children’s Hospital, Harvard Medical School, Harvard Institutes of Medicine, Boston Massachusetts 02115, USA

^2^Department of Surgery, Boston Children’s Hospital, Harvard Medical School, Boston, Massachusetts 02115, USA

^3^Department of Anesthesiology, Boston Children’s Hospital, Harvard Medical School, Boston, Massachusetts 02115, USA

*Corresponding author

E-mail: daniel.kohane@childrens.harvard.edu

^¶^These authors contributed equally to this work

**SI Figure 1**


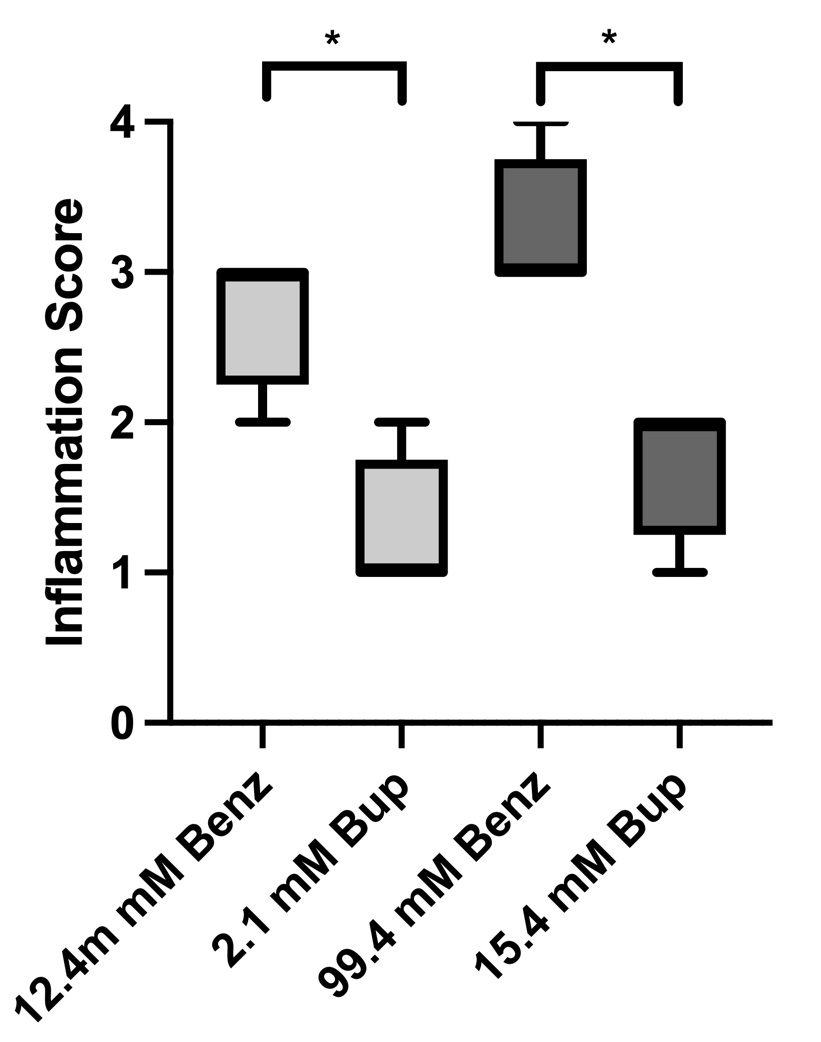

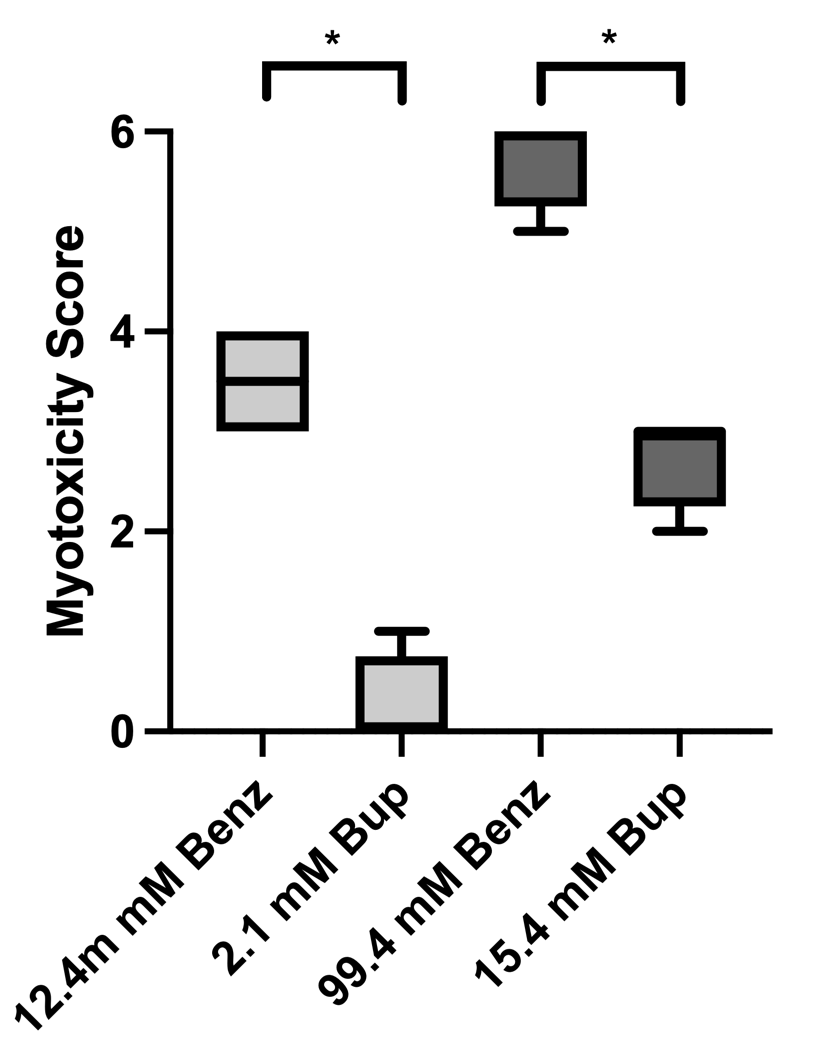


**Figure S1.** Quantification of tissue reaction represented by a box and whisker plot. (a) Inflammation and (b) myotoxicity scores of animals injected at the sciatic nerve with benzonatate and bupivacaine. Equieffective concentrations have the same shading. Data are medians with 25^th^ and 75^th^ percentiles, n=4 for all groups. Note that the median value (bar) overlaps with the 25^th^ or 75^th^ percentiles (box) for multiple data points. * P < 0.05 for the comparison of equieffective concentrations by Mann-Whitney U test.
