# Supplementary material for: Benzonatate as a local anesthetic
Source: PLoS One. 2023 Apr 12;18(4):e0284401. doi: 10.1371/journal.pone.0284401 (PMC10096445; doi:10.1371/journal.pone.0284401)
Supplement: S1 Table — (DOCX) [file pone.0284401.s002.docx]

**SI Table 1. Formulations injected at the sciatic nerve.**

| **Benzonatate (mM)** | **Co-administered with** | **Animal Number** | **Sensory Block Duration (min)** | **Motor Block Duration (min)** |
| --- | --- | --- | --- | --- |
| 5.4 | - | Animal #1 | 0 | 42.9 |
|  |  | Animal #2 | 74.0 | 69.7 |
|  |  | Animal #3 | 0 | 44.6 |
|  |  | Animal #4 | 0 | 0 |
| 12.4 | - | Animal #1 | 0 | 0 |
|  |  | Animal #2 | 77.4 | 100.7 |
|  |  | Animal #3 | 44.2 | 45.7 |
|  |  | Animal #4 | 0 | 41.9 |
|  |  | Animal #5 | 47.3 | 81.7 |
|  |  | Animal #6 | 81.3 | 84.2 |
|  |  | Animal #7 | 0 | 51.5 |
|  |  | Animal #8 | 76.4 | 109.1 |
|  |  | Animal #9 | 0 | 0 |
|  |  | Animal #10 | 50.7 | 52.4 |
|  |  | Animal #11 | 85.3 | 102.8 |
|  |  | Animal #12 | 61.1 | 66.2 |
| 24.8 | - | Animal #1 | 0 | 0 |
|  |  | Animal #2 | 104.5 | 105.9 |
|  |  | Animal #3 | 64.5 | 76.4 |
|  |  | Animal #4 | 137.3 | 153.1 |
| 49.7 | - | Animal #1 | 151.3 | 147.1 |
|  |  | Animal #2 | 148.5 | 151.0 |
|  |  | Animal #3 | 151.6 | 154.2 |
|  |  | Animal #4 | 89.5 | 98.2 |
| 74.5 | - | Animal #1 | 159.7 | 156.4 |
|  |  | Animal #2 | 134.5 | 131.8 |
|  |  | Animal #3 | 89.7 | 85.7 |
|  |  | Animal #4 | 135.4 | 129.4 |
| 99.4 | - | Animal #1 | 148.9 | 189.7 |
|  |  | Animal #2 | 186.0 | 151.6 |
|  |  | Animal #3 | 148.2 | 152.5 |
|  |  | Animal #4 | 148.5 | 139.5 |
|  |  | Animal #5 | 166.2 | 168.2 |
|  |  | Animal #6 | 165.9 | 167.4 |
|  |  | Animal #7 | 166.2 | 168.6 |
|  |  | Animal #8 | 136.2 | 135.5 |
| 149.1 | - | Animal #1 | 198.2 | 224.9 |
|  |  | Animal #2 | 197.6 | 224.8 |
|  |  | Animal #3 | 196.5 | 227.3 |
|  |  | Animal #4 | 196.6 | 196.7 |
| 198.8 | - | Animal #1 | 237.4 | 322.9 |
|  |  | Animal #2 | 195.9 | 231.8 |
|  |  | Animal #3 | 221.5 | 228.8 |
|  |  | Animal #4 | 229.2 | 466.3 |
| - | 30 μM TTX | Animal #1 | 0 | 0 |
|  |  | Animal #2 | 0 | 0 |
|  |  | Animal #3 | 0 | 0 |
|  |  | Animal #4 | 0 | 0 |
|  |  | Animal #5 | 0 | 0 |
|  |  | Animal #6 | 49.2 | 76.9 |
|  |  | Animal #7 | 49.6 | 46.6 |
|  |  | Animal #8 | 0 | 38.6 |
| 12.4 | 30 μM TTX | Animal #1 | 77.9 | 76.3 |
|  |  | Animal #2 | 176.3 | 325.0 |
|  |  | Animal #3 | 74.7 | 89.0 |
|  |  | Animal #4 | 77.8 | 80.2 |
|  |  | Animal #5 | 47.0 | 75.2 |
|  |  | Animal #6 | 74.5 | 77.6 |
|  |  | Animal #7 | 130.2 | 165.1 |
|  |  | Animal #8 | 222.0 | 226.3 |
| - | 23 mM T80 | Animal #1 | 0 | 0 |
|  |  | Animal #2 | 0 | 0 |
|  |  | Animal #3 | 0 | 0 |
|  |  | Animal #4 | 0 | 0 |
|  |  | Animal #5 | 0 | 0 |
|  |  | Animal #6 | 0 | 0 |
|  |  | Animal #7 | 0 | 0 |
|  |  | Animal #8 | 0 | 0 |
| 12.4 | 23 mM T80 | Animal #1 | 75.5 | 99.7 |
|  |  | Animal #2 | 104.5 | 132.0 |
|  |  | Animal #3 | 74.6 | 105.1 |
|  |  | Animal #4 | 76.4 | 106 |
|  |  | Animal #5 | 110.5 | 141.5 |
|  |  | Animal #6 | 106.0 | 137.8 |
|  |  | Animal #7 | 77.5 | 105.3 |
|  |  | Animal #8 | 76.3 | 108 |

- Indicates that agent was not administered
