# Supplementary material for: Benzonatate as a local anesthetic
Source: PLoS One. 2023 Apr 12;18(4):e0284401. doi: 10.1371/journal.pone.0284401 (PMC10096445; doi:10.1371/journal.pone.0284401)
Supplement: S2 Table — (DOCX) [file pone.0284401.s003.docx]

**SI Table 2. Tissue Toxicity of Benzonatate: Inflammation and Myotoxicity Scores.**

| **Compound** | **Concentration (mM)** | **Sample Number** | **Inflammation Score** | **Myotoxicity Score** |
| --- | --- | --- | --- | --- |
| Benzonatate | 12.4 | Sample #1 | 2 | 3 |
|  |  | Sample #2 | 3 | 4 |
|  |  | Sample #3 | 3 | 4 |
|  |  | Sample #4 | 3 | 3 |
| Bupivacaine | 2.1 | Sample #1 | 1 | 0 |
|  |  | Sample #2 | 1 | 0 |
|  |  | Sample #3 | 1 | 0 |
|  |  | Sample #4 | 2 | 1 |
| Benzonatate | 99.4 | Sample #1 | 3 | 5 |
|  |  | Sample #2 | 3 | 6 |
|  |  | Sample #3 | 4 | 6 |
|  |  | Sample #4 | 3 | 6 |
| Bupivacaine | 15.4 | Sample #1 | 2 | 3 |
|  |  | Sample #2 | 2 | 3 |
|  |  | Sample #3 | 1 | 2 |
|  |  | Sample #4 | 2 | 3 |
